# Transportation of Wounded

**Published:** 1918-09

**Authors:** R. S. C. Thompson


**Transportation of Wounded.** By Lt.-Colonel R. S. C. Thompson, R. A. M. C.

Considering the transportation of wounded by motor ambulance the speaker stated that three important points present themselves :

1. Distance.
2. Speed.
3. Welfare of patients during the journey.

1. *Distance.* Preference has been given to the transportation of severe cases such as abdominal or chest wounds to a medical unit where they can be fully and completely dealt with under the best available conditions. This is safer than to attempt their treatment in proximity to the line.

As a rule these units are situated at a distance of anything from eight to fifteen miles from the front line, and the speaker thinks that there is no doubt that the longer journey is compensated for by the installation of the patient in a complete medical unit out of range and sound of the battle field rather than in the atmosphere of uncertainty and anxiety prevailing in an operating center within two or three miles of the line.

2. *Speed of Vehicles.* This must be determined to a great extent by the discretion of the drivers founded upon a knowledge of the peculiarities of their own particular cars. In any case, however, it cannot be too strongly impressed upon all concerned that the transport of badly wounded men is a matter calling for the utmost care and skill.

Jolting over a road which is full of holes must necessarily cause acute pain to patients with fractures while it may also provoke or re-awaken fatal hemorrhage. In chest wounds, for instance, Duval in his book says : — " Patients die or have fatal hemorrhage, not only during transport, but because of the transport ". In this connection, the roads along which the ambulances are destined to pass should be examined beforehand, and drivers should also be prepared and trained to drive without lights in case this should be necessary.

3. *Welfare of Patient During the Transport.* The aggravation of surgical shock should be reduced as much as possible as this is a very important point. To this end the A. D. S.'s and the C. C. S.'s must act in close co-operation so that the two units work together as though they were part of the same organism controlled by one brain.

It is exceedingly important that the patient should be made warm for the journey with blankets and hot-water bottles. It is equally so that he should be given primary treatment for shock, and except in times of stress should not be transferred until considered to be in a fit condition to stand the journey.

Hemorrhage should be checked. If a tourniquet has been considered necessary, this fact must be so clearly marked that the receiving unit cannot fail to know of it as soon as the patient arrives.

Fractures must be immobilized before the patient is placed on the car.

The bladder should be emptied before the journey begins, and the administration of morphia governed by circumstances.

An orderly should travel inside each car conveying dangerously wounded patients.

Severed limbs should be completely amputated before starting.

Bitter experience has taught that the most careful organization is necessary to ensure that the patient's small belongings are transported with him, and for this purpose no advance unit can afford to run short of Lady Smith Dorrien's treasure bags.

When the question of refreshment during the journey is considered, tea receives 99 o/o preference above coffee, cocoa, or soup.

With regard to " stored blood " for indirect transfusion, in many places it has been found difficult to store it in a cold enough place, and it seems that a properly prepared 6 o/o gum acacia solution in normal saline is the next best material for intravenous infusion.

A central laboratory for the turning out of the gum acacia solution in well-stoppered sterilized bottles is essential, in view of the unreliability of the water supply.

Some wounds to be specially considered from the transport point of view are :

1. *Wounds of the Eye.* In the case of a f. b. the patient must be conveyed as quickly as possible to the unit where ophthalmic surgeons are available. In case, however, of complications of a different and more dangerous nature, it should be possible to retain the patient in the C. C. S. and to have him visited there by a mobile ophthalmic surgeon available by telephone, equipped with a magnet and cable adjustable to the C. C. S. electric plant.

2. *Fractures.* — The speaker believed in a minimum variety of splints, and in the advanced units and the C. C. S.'s being word-perfect in their use.

For wounds with involvement of joints, use a Thomas splint. If the buttock or region of the great trochanter is involved, the interrupted Liston should be used. The iron skewer through the boot is the quickest adjunct to the Thomas splint, less gangrene of the foot following its use. Remember to place a second suspension bar over the patient's hip, so that he can use it during the trip to lessen the vibration of the car. Sinclair has recently made one which can be adjusted on the inside of the wooden side members of a stretcher for the suspension of the foot and of the Thomas splint.

For a fracture of the upper extremity the author considers that a Thomas swivel arm splint is the best. Failing Thomas' leg splint, the attachment of feet to stretcher and perineal sling. *Loosen all ties* as soon as possible. In the case of an open thorax, the sucking pneumothorax must be closed at the earliest opportunity, and, if not sutured, the pad closing the wound removed as early as possible. Sutures closing the chest are infinitely preferable to the firm application of a pad over the hole in the chest.

Gassed cases should always travel as lying-down cases on a stretcher.

Each C. C. S. should have a road or roads by which reception of patients and evacuation to ambulance train can be carried on at the same time. It is necessary to have in the C. C. S.: (1) A special party of stretcher bearers for each stretcher bearing service. (2) A sufficiently large reception room or annex. (3) A means by which the drivers of the cars can pick up an equivalent number of stretchers, blankets, and so forth, to that which they have brought with the patients. At times the C. C. S. must even supply exhausted and under-equipped A. D. S. stores of blankets, and a considerable reserve must be held.

The ideal is for the C. C. S. to disinfect all blankets arriving from the A. D. S. The disinfection of cars after conveying an infectious case is best carried out directly the case arrives at its destination.

